# Gemcitabine Maintenance Therapy in Patients With Metastasized Soft Tissue Sarcomas

**DOI:** 10.3389/fonc.2021.755439

**Published:** 2021-12-14

**Authors:** Dennis Christoph Harrer, Sebastian Buschauer, Ulrich Sterz, Karin Menhart, Christina Wendl, Daniel Heudobler, Matthias Grube, Tobias Pukrop, Wolfgang Herr, Martin Vogelhuber

**Affiliations:** ^1^ Department of Medicine III—Hematology and Oncology, University Hospital Regensburg, Regensburg, Germany; ^2^ Department of Nuclear Medicine, University Hospital Regensburg, Regensburg, Germany; ^3^ Department of Radiology, University Hospital Regensburg, Regensburg, Germany

**Keywords:** sarcoma, maintenance therapy, solid tumor, chemotherapy, stroma tumor

## Abstract

**Background:**

Metastasized soft-tissue sarcomas still pose a significant therapeutic challenge given the limited efficacy of currently available multimodal treatment strategies. Recent progress in molecular characterization of sarcoma subtypes has enabled successful personalized therapy approaches in a minority of selected patients with targetable mutations. However, in the majority of patients with refractory soft tissue sarcomas, long-term survival remains poor.

**Methods:**

We report on three adult patients with various soft tissue sarcomas subjected to Gemcitabine maintenance therapy. Tumor entities included leiomyosarcoma of the pancreas (patient 1), undifferentiated pleomorphic sarcoma of the right femur (patient 2), and peri-aortic leiomyosarcoma (patient 3). Metastatic sites encompassed liver, lung, and bones. All patients received Gemcitabine maintenance therapy until disease progression following prior salvage chemotherapy with Docetaxel and Gemcitabine. Patients were treated outside of clinical trials. Response assessment was based on radiological imaging.

**Results:**

In response to salvage chemotherapy with Docetaxel and Gemcitabine, one patient exhibited a partial remission, and two patients showed stable disease. Patient 1 exhibited stable disease for 6 months during Gemcitabine maintenance therapy before suffering rapid progression of hepatic metastases. Patient 2 underwent 21 months of Gemcitabine maintenance therapy, which was discontinued after progressive pulmonary metastases were detected. Patient 3 is still being treated with Gemcitabine maintenance therapy. Remarkably, owing to significant chemotherapy-associated hematotoxicity, the dose of Gemcitabine dose was reduced by two-thirds. Nevertheless, stable disease with constant pulmonary metastases has been maintained in this patient for 14 months.

**Conclusions:**

Gemcitabine maintenance therapy following prior Docetaxel and Gemcitabine chemotherapy is manageable and reveals potential benefits for patients with aggressive metastasized soft tissue sarcomas. Prospective trials evaluating Gemcitabine maintenance therapy are encouraged.

## Background

Despite multiple new anticancer agents available, the long-term survival of patients with advanced sarcomas remains dismal (1, 2). Soft tissue sarcomas (STS) represent a heterogeneous class of rare tumors originating from different soft connective tissues, such as smooth muscle or fat (3). Overall, STS comprise <1% of all adult malignancies amounting to 12,000 new cases per year in the US (2). While the majority of STS is located in the extremities, they can arise from virtually any site of the body (3). Metastatic spread occurs frequently into the lungs followed by the liver and bones (3). Distant metastases are associated with a bad prognosis reflected by 5-year survival rates of about 15%, whereas patients with local STS exhibit 5-year survival of approximately 80% (2). The paramount prognostic factors are the histological subtype, tumor–node–metastasis (TNM) stage, and surgical resection margins (2).

Current treatment strategies adopt a multimodal approach combining surgery, radiotherapy, chemotherapy, and targeted therapy (2, 4). Chemotherapy agents with efficacy in soft tissue sarcomas encompass Doxorubicin (5), Ifosfamide (6), Gemcitabine (7), Docetaxel (8), Dacarbazine (9), Eribulin (10), and Trabectedin (11). Targeted therapy approaches include tyrosine kinase inhibitors, such as the Pazopanib and the multikinase inhibitor Regorafenib (12, 13). In case of detectable fusion proteins involving neurotrophic receptor tyrosine kinase (NTRK) 1–3 present in <1% of all STS (14), second-line treatment with the NTRK inhibitor Larotrectinib could mediate tumor regression in the majority of patients with 16% of complete responders (15). The monoclonal antibody Olaratumab directed against the platelet-derived growth factor alpha (PDGFR-alpha) was initially approved for STS in combination with Doxorubicin but was later withdrawn from the market due to lacking efficacy in a phase III clinical trial (16). In aggregate, specific treatment strategies are devised based on histological subtypes, disease stage, and patient condition. Currently, a multitude of histologic subtypes of STS has been described (17).

Malignant tumors arising from smooth muscle tissue are denoted as leiomyosarcomas and account for 10% of all STS, making them one of the most common subtype in adults (18). Predilection sites for leiomyosarcomas are the extremities followed by the uterus, small intestine, and retroperitoneal space (19). Given an insufficient sensitivity to chemotherapy and radiation, surgical excision with wide resections margins constitutes the standard of care in non-uterine leiomyosarcoma treatment (20, 21). Relapse after resection or primary irresectable disease bodes prognostically ill, with 5-year survival rates approximately 10–15% (2, 22). Chemotherapeutic agents with activity in advanced leiomyosarcoma include Doxorubicin (5), Ifosfamide (6), Dacarbazine (9), and Trabectedin (23). Moreover, monotherapy with Pazopanib demonstrated clinically relevant efficacy and tolerability in patients with leiomyosarcoma (12).

In patients with metastatic/advanced STS, systemic therapy aims at reducing tumor burden, thereby alleviating symptoms and improving quality of life (24). However, many STS exhibit primary or acquired resistance to chemotherapy resulting in only short periods of tumor control achieved by systemic treatment (25). Moreover, the toxicity/efficacy ratio of potentially aggressive systemic treatments should be considered with regards to maintaining quality of life during this palliative treatment. Thus, continuation of systemic therapy with dose-reduced maintenance therapy intended to preserve beneficial responses (complete or partial response or stable disease) seems worth exploring in patients with STS. In general, maintenance therapy aims at slowing down disease progression and at improving quality of life. In clinical practice, maintenance therapy could be administered either until disease progression or until the occurrence of major toxicity. As for patients with metastatic/advanced soft tissue sarcomas, there is currently no recommended maintenance therapy, apart from combination maintenance therapy with Cyclophosphamide and Vinorelbine in localized high-risk rhabdomyosarcoma (24, 26). Moreover, only few studies evaluating the efficacy of maintenance therapy in patients with STS have been conducted (24).

In this study, we present a retrospective analysis of three patients with metastasized STS who underwent maintenance therapy with Gemcitabine on an off-label basis at the University Hospital of Regensburg. Gemcitabine maintenance therapy administered after prior Docetaxel and Gemcitabine chemotherapy showed potential to slow down tumor progression in all three patients without compromising quality of life to a significant extent.

## Methods

Sarcoma diagnosis was confirmed in all three patients by histological examination [hematoxylin and eosin (H&E) staining and immunohistochemistry] and molecular analysis (fluorescence *in situ* hybridization) of biopsies obtained *via* excision or imaging-guided puncture. Tumor response was assessed by computed tomography (CT), magnetic resonance imaging (MRI), and F-18-fluorodeoxyglucose–positron emission tomography (PET)/computed tomography (CT). Patients were treated at the Sarcoma Center of the University Hospital of Regensburg between 2014 and 2021. All patients received treatment independent of clinical trials. Data analysis was carried out retrospectively. The study was approved by the Ethics Committee of the University Regensburg (ethics statement no. 21-2532-104). Initial Gemcitabine dosing varied from 900 mg/m^2^ (d1 and d8 with repetition d22) to 500 mg/m^2^ (d1, d15 with repetition d29) and was individually adapted during the course of treatment (**Table 1**). Clinical outcomes were tracked until June of 2021. Written, informed consent to publishing was obtained.

**Table 1:** Gemcitabine maintenance therapy regimen.

Pat. ID	Dose^a^	Days in cycle	Repetition	Comments
1	900 mg/m^2^	d1, d8	d22	Reduced dose due to leukopenia
2	900 mg/m^2^	d1, d8	d22	Reduced dose due to leukopenia
3	500 mg/m^2^	d1, d15	d29	Initial dose of 1,000 mg/m^2^ but dose reduction for all subsequent cycles due to excessive hematotoxicity

a Corresponding absolute doses calculated based on adjusted ideal body weight (AIBW).

## Results

**Figure 1:**
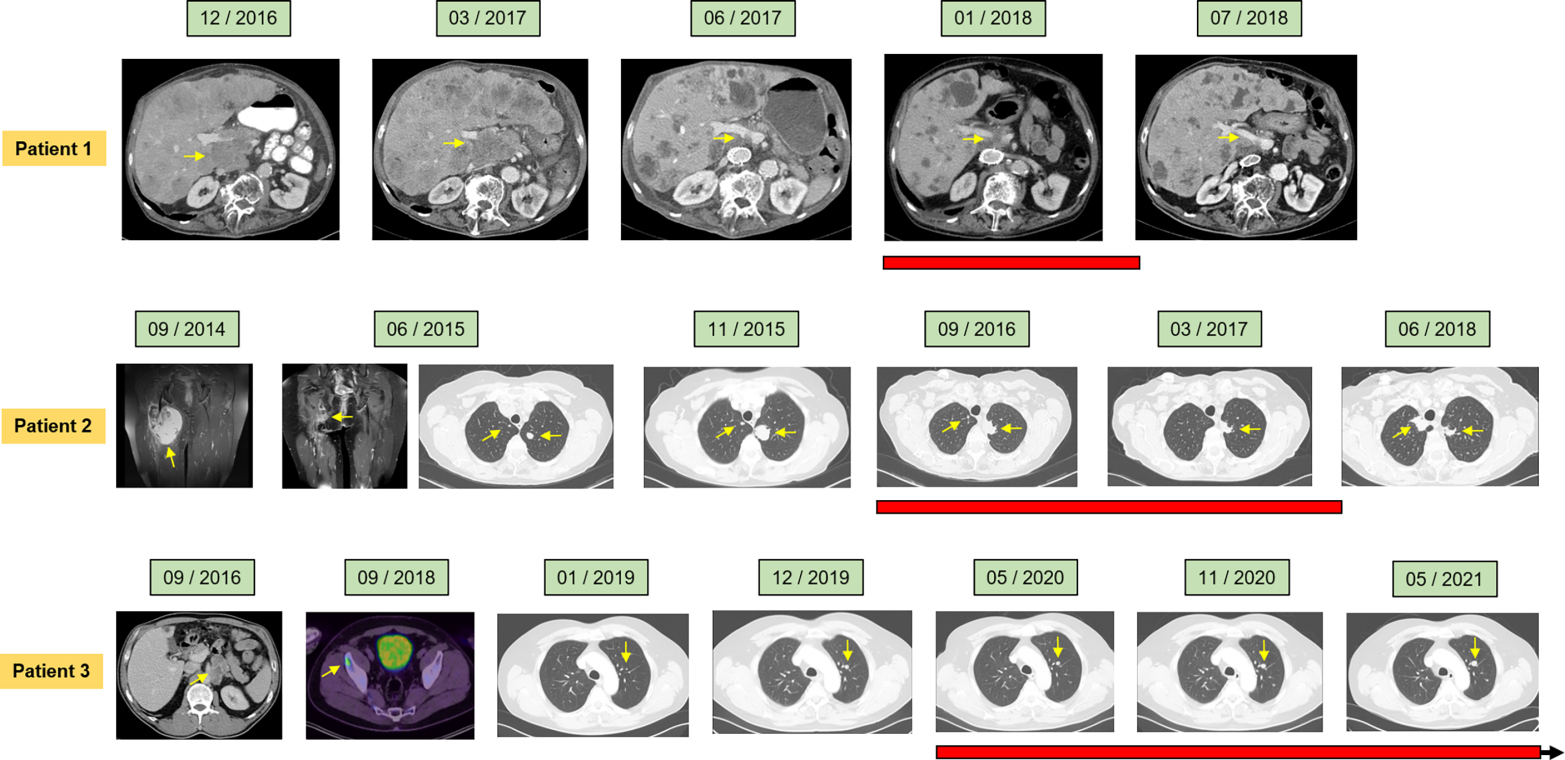
Clinical course of the individual patients assessed by radiological imaging depicting treatment response at the indicated time points. Computed tomography (CT), magnetic resonance imaging (MRI), and positron emission tomography were utilized (PET). Red bars denote duration of Gemcitabine maintenance therapy. Arrows indicate ongoing Gemcitabine maintenance therapy.

**Figure 2:**
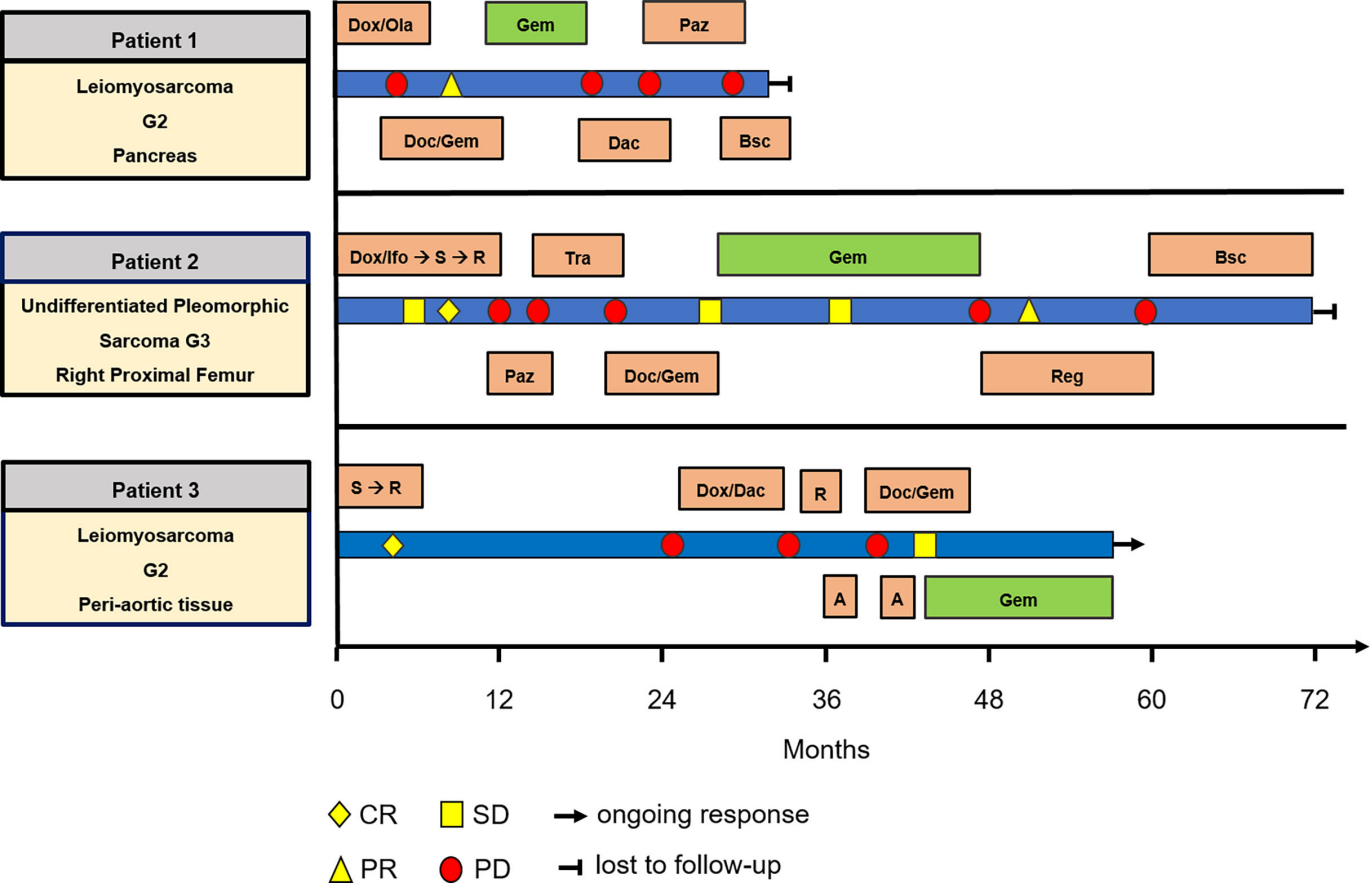
Swimmer plot including all patients, showing treatment and response. A, ablation; Bsc, best supportive care; Dac, Dacarbazine; Doc, Docetaxel; Dox, Doxorubicin; Gem, Gemcitabine; Ifo, Ifosfamide; Ola, Olaratumab; Paz, Pazopanib; R, radiation; Reg, Regorafenib; S, surgery; Tra, Trabectedin.

*Patient 1. A* 73-year-old male patient diagnosed with moderately differentiated leiomyosarcoma of the pancreas and multiple hepatic metastases was referred to our Department of Hematology and Oncology at the University Hospital of Regensburg (**Table 2** and **Figure 1**). The patient received two cycles of Doxorubicin in combination with the PDGFR alpha blocking monoclonal antibody Olaratumab. However, magnetic resonance imaging (MRI) of the liver performed after 3 months of therapy showed rapid tumor progression in both the liver and the pancreas (**Figure 1**). Therefore, chemotherapy was switched to Gemcitabine combined with Docetaxel. In response to four cycles of treatment with Gemcitabine/Docetaxel remission control *via* liver MRI revealed partial remission with shrinking hepatic metastases and reduction in tumor size in the pancreas (**Figure 1**). Owing to considerable residual tumor volume and initial aggressive tumor growth refractory to first-line chemotherapy, the patient was individually selected for maintenance therapy with Gemcitabine, which was started 6 months after the first cycle of Gemcitabine/Docetaxel. Originally, dosing of Gemcitabine during maintenance therapy was supposed to be 1,000 mg/m^2^ on days 1, 8, and 15 with cycle repetition every 3 weeks. Nevertheless, due to previous hematotoxicity (prolonged neutropenia) experienced during Gemcitabine/Docetaxel treatment, the Gemcitabine dose was reduced to 900 mg/m^2^ in this patient administered on days 1 and 8 with repetition every 3 weeks (**Table 1**). No serious complications occurred during Gemcitabine monotherapy. Moreover, based on medical history records regularly updated on gemcitabine infusion appointments, quality of life was not severely impaired. Six months after the start of gemcitabine maintenance therapy, progressive disease was detected by liver MRI. Apart from an increase in size of known metastases, several new hepatic lesions appeared. Salvage therapy was first attempted with Dacarbazine monotherapy and, due to lacking efficacy, was switched to Pazopanib 3 months later. Unfortunately, disease progression could not be hampered by Pazopanib either, and in the face of beginning acute liver failure, palliative care was initiated, and the patient died in the following month, 2 1/2 years after the initial diagnosis of leiomyosarcoma. In aggregate, Gemcitabine/Docetaxel combination therapy with subsequent Gemcitabine maintenance therapy could induce partial tumor regression and slow tumor growth for several months in this patient, who suffered from rapid progressive leiomyosarcoma refractory to Doxorubicin, Dacarbazine, and Pazopanib (**Figure 2**).

**Table 2:** Patient characteristics and response to Gemcitabine maintenance therapy.

Pat. ID	Sex	Age^a^	Disease	Metastases	Pretreatment	Duration^b^	Best Response^c^
1	Male	73y	Leiomyosarcoma of pancreas G2	Liver, Lung	Doxorubicin/Olaratumab Docetaxel/Gemcitabine	6 months	SD (6 months)
2	Female	61y	Pleomorphic sarcoma of right femur G3	Lung	Doxorubicin/Ifosfamide	21 months	SD (21 months)
					Surgery		
					Radiation		
					Pazopanib		
					Trabectedin		
					Local ablation		
					Docetaxel/Gemcitabine		
3	Male	54y	Peri-aortic Leiomyosarcoma G2	Liver, Lung, Bones	Surgery	14 months ongoing	SD (14 months, ongoing)
					Radiation		
					Doxorubicin/Dacarbazine		
					Local ablation		
					Gemcitabine/Docetaxel		

a At first diagnosis.

b Gemcitabine maintenance therapy.

c Response to gemcitabine maintenance therapy.

SD, stable disease.

*Patient 2. A* 61-year-old female patient presented with an undifferentiated pleomorphic sarcoma of the right femur (**Table 2**). Radiological examination *via* MRI revealed a pseudoaneurysmatic tumor protruding from the back of the right femur (**Figure 1**). The original treatment strategy included surgical removal after prior chemotherapy. Accordingly, the patient received neoadjuvant chemotherapy with Doxorubicin and Ifosfamide. After two cycles of chemotherapy, however, no significant tumor regression could be achieved. Hence, a decision to surgically remove the tumor with subsequent radiation of the resection site was made. Sarcoma removal was performed at the Sarcoma Center of the University Hospital of Regensburg. After complete excision with no residual tumor cells on the resection margin (R0), the patient underwent adjuvant radiation therapy. Thereafter, regular follow-up was started. Unfortunately, 3 months after regular follow-up, MRI imaging showed a contrast-enhancing lesion at the upper resection margin (**Figure 1**). Besides, pulmonary CT imaging showed several lung metastases (**Figure 1**). Relapse therapy with Pazopanib was started, but no significant inhibition of growth of the pulmonary metastases could be attained necessitating third-line therapy with Trabectedin, which did not achieve any remission either (**Figure 1**). Analogous to patient 1, salvage therapy was attempted with the combination of Gemcitabine and Docetaxel. In response to eight cycles of Gemcitabine/Docetaxel treatment, the patient showed stable pulmonary metastases without any new or growing lesions. Equal to patient 1, residual tumor masses and the aggressive tumor biology with insensitivity to several different anti-sarcoma drugs prompted the start of Gemcitabine maintenance. Similar to patient 1, prior hematotoxicity (neutropenia) suffered during Gemcitabine/Docetaxel treatment rationalized a reduced dose of 900 mg/m^2^ in this patient, administered on days 1 and 8 with repetition every 3 weeks (**Table 2**). Six months after Gemcitabine maintenance therapy, pulmonary CT imaging showed stable disease with constant bipulmonary metastases (**Figure 1**). During Gemcitabine maintenance therapy, no serious complications were documented, and quality of life was not impaired in this patient, as assessed at doctor’s appointments on chemotherapy infusion days. After 21 months of Gemcitabine therapy, the patient showed increasing pulmonary metastases resulting in cessation of Gemcitabine treatment (**Figure 1**). Salvage therapy with Regorafenib was started, which could induce a partial remission after 2 months of treatment, which lasted for 1 year. Given no further treatment options available, best supportive care was commenced. The patient died 1 year later, nearly 5 years after the initial diagnosis of leiomyosarcoma. In aggregate, Gemcitabine/Docetaxel combination therapy with subsequent Gemcitabine maintenance therapy appeared to slow down tumor growth for more than 1 year in this patient, who displayed aggressive undifferentiated pleomorphic sarcoma refractory to Doxorubicin, Ifosfamide, Pazopanib, and Trabectedin (**Figure 2**).

*Patient 3. A* 54-year-old man presented with moderately differentiated peri-aortic leiomyosarcoma (**Table 2**). No metastases were present at initial diagnosis. The treatment strategy encompassed complete surgical excision followed by adjuvant radiotherapy. After successful surgical excision (R0 resection), adjuvant radiotherapy was started. Afterwards, regular follow-up examinations were initiated. Two years later, a metabolically active lesion was detected in the right ilium *via* PET-CT (**Figure 1**). Biopsy and histological examination revealed leiomyosarcoma relapse. Chemotherapy with Dacarbazine and Doxorubicin was administered. Under this therapy, increasing pulmonary metastases and new liver lesions were observed, indicating refractory disease. Subsequently, the osseous metastasis was irradiated, liver lesions were locally ablated, and analogous to patients 1 and 2, salvage therapy with Gemcitabine and Docetaxel was started. After two cycles of Gemcitabine/Docetaxel treatment, the patient exhibited stable disease and subsequently was put on Gemcitabine maintenance therapy to slow down further tumor progression. Patient 3 initially received three fully dosed cycles with 1,000 mg/m^2^ Gemcitabine on days 1, 8, and 15 (**Table 1**). However, severe prolonged neutropenia prompted first omission of day 8 and pegfilgrastim support on days 2 and 16 with cycle repetition on day 29. Later during the sixteenth cycle of Gemcitabine maintenance therapy, persistent neutropenia necessitated dose reduction in Gemcitabine to 500 mg/m^2^ administered every 2 weeks (**Table 1**). At 6 months after start of Gemcitabine monotherapy, the patient maintained stable disease with size constant pulmonary metastases (**Figure 1**). Until now, the patient has completed 14 months of Gemcitabine maintenance therapy without contracting any serious complications or reduction in quality of life. Just recently (May 2021), stable disease was reconfirmed *via* CT imaging (**Figure 1**). The patient is scheduled to continue Gemcitabine maintenance therapy until tumor progression or the occurrence of unbearable side effects. In aggregate, Gemcitabine/Docetaxel combination therapy with subsequent Gemcitabine maintenance therapy appeared to slow down tumor growth for more than 1 year in this patient, who displayed aggressive leiomyosarcoma refractory to Doxorubicin and Dacarbazine (**Figure 2**). Interestingly, this patient remains stable even with a significantly reduced Gemcitabine dosing, which allows for continuation of Gemcitabine maintenance therapy.

## Discussion

In patients with metastasized soft tissue sarcomas, long-term survival is rare, and effective treatment strategies are lacking (15). Particularly for patients with refractory or relapsed soft tissue sarcomas, there is an urgent need to enhance the efficacy of salvage chemotherapy. The concept of maintenance therapy aims at delaying disease progression and improving quality of life. In this case series, we retrospectively analyzed the concept of maintenance therapy in patients with different soft tissue sarcomas including leiomyosarcoma of the pancreas, undifferentiated pleomorphic sarcoma of the femur, and peri-aortic leiomyosarcoma. After salvage therapy with Gemcitabine and Docetaxel, Gemcitabine maintenance therapy in an off-label fashion was given, aiming to slow down disease progression in patients with aggressive soft tissue sarcomas.

We report promising therapeutic potential for Gemcitabine maintenance therapy even after significant dose reductions in Gemcitabine due to hematotoxicity. All patients in this case series suffered from aggressive metastasized STS refractory to at least one line of chemotherapy.

During Gemcitabine maintenance therapy, two patients showed stable disease lasting several months, and one patient exhibited partial disease regression maintained for 21 months. These favorable results could be achieved despite individual adaption of Gemcitabine dose according to comorbidities and pretreatment.

All patients included in this study were treated individually and independently from one another. Hence, individual treatment regimens differ; thus, direct comparability of patients is compromised, also the generation of pooled analyses for usual outcome parameters such as overall survival and progression-free survival. Further limitations to this study include the non-interventional retrospective design of this analysis and the limited number of patients allowing only a narrative description of the individual courses.

In general, data about the clinical benefit of maintenance therapy in sarcoma are scant (24). Only in patients with localized high-risk rhabdomyosarcoma combination maintenance therapy with Cyclophosphamide and Vinorelbine administered over 6 months could significantly improve overall survival (26). In contrast, maintenance therapy with both anti-PDGFR alpha blocking monoclonal antibody Olaratumab and the vascular endothelial growth factor antagonist Bevacizumab failed to significantly slow down disease progression (16, 27). In addition, the mammalian target of rapamycin (mTOR) inhibitor ridaforolimus (RIDA) significantly delayed disease progression in patients with advanced soft tissue sarcoma but did not significantly improve overall survival (28). In conclusion, the sole clinical setting in which maintenance therapy in soft tissue sarcoma is supported by positive clinical data is represented by high-risk localized rhabdomyosarcoma.

Furthermore, the toxicity profile of maintenance therapy must be contemplated carefully. In case of advanced disease, maintenance therapy regimens administered to delay disease progression must be well tolerated without significant impairments of quality of life. In all three patients presented, quality of life was not impaired by Gemcitabine maintenance therapy. Moreover, Gemcitabine is a generally well-tolerated chemotherapeutic agent, with hematotoxicity being the most important major side effect (29). Hence, especially with regards to the Gemcitabine/Docetaxel pretreatment, Gemcitabine maintenance therapy was individually adjusted based on condition and bone marrow function of the individual patients. Therefore, the targeted dose of 1,000 mg/m^2^ was slightly reduced in two patients to 900 mg/m^2^ and in one patient to 500 mg/m^2^. Despite the significant dose reduction in Gemcitabine in patient 3, the efficacy in terms of tumor progression was not affected. Thus, Gemcitabine maintenance therapy might also be suitable for elderly patients and patients with heavy pretreatment, as it can be used in reduced dose in case of marrow dysfunction.

Only a few effective chemotherapy regimens are currently available for treatment of advanced soft tissue sarcoma, and there have been very few phase III clinical trials of second-line chemotherapy. All patients in this study were progressive to at least one line of systemic therapy. Before initiation of Gemcitabine maintenance therapy, all patients received salvage therapy with Gemcitabine and Docetaxel. In a large retrospective study reported in 2006 analyzing 133 patients with soft tissue sarcoma from 10 institutions in France, the overall response rate to Gemcitabine and Docetaxel combination therapy was 18.4%, and the median overall survival was 12.1 months (30). Similarly, in a Korean study covering 281 patients, the objective response rate was 15.6%, while the median overall survival was 10.3 months (31). In contrast, a phase 2 clinical trial evaluating second-line therapy with gemcitabine monotherapy in patients with various sarcomas showed an objective response rate of only 5.5% (32). Based on the data of those studies, Gemcitabine/Docetaxel therapy was selected as salvage treatment. Finally, we suggest using Gemcitabine/Docetaxel prior to Gemcitabine maintenance therapy because an objective response achieved by Gemcitabine/Docetaxel might indicate increased chances of sensitivity to Gemcitabine chemotherapy.

In summary, we would like to raise awareness for the concept of Gemcitabine maintenance therapy as novel therapeutic modality in patients suffering from soft tissue sarcoma with special emphasis on patients with aggressive disease refractory to prior chemotherapy. In three patients with soft tissue sarcoma, aggressive disease progression could be slowed down with Gemcitabine maintenance therapy. Based on those remarkable results obtained from individual off-label treatments, we encourage the initiation of early phase clinical trials to prospectively evaluate Gemcitabine maintenance therapy in patients with soft tissue sarcomas.

## Data Availability Statement

The data analyzed in this study is subject to the following licenses/restrictions: the datasets underlying this study are available on request to the corresponding author. Requests to access these datasets should be directed to dennis.harrer@ukr.de.

## Ethics Statement

The studies involving human participants were reviewed and approved by Ethics Committee of the University Regensburg. The patients/participants provided their written informed consent to participate in this study.

## Author Contributions

MV conceptualized the study. MV, US, DH, MG, and SB treated the patients. DCH drafted the manuscript. KM provided the positron emission imaging material. CW provided the CT and MRI material. WH, TP, and MG interpreted data and critically revised the manuscript. All authors contributed to the article and approved the submitted version.

## Conflict of Interest

The authors declare that the research was conducted in the absence of any commercial or financial relationships that could be construed as a potential conflict of interest.

## Publisher’s Note

All claims expressed in this article are solely those of the authors and do not necessarily represent those of their affiliated organizations, or those of the publisher, the editors and the reviewers. Any product that may be evaluated in this article, or claim that may be made by its manufacturer, is not guaranteed or endorsed by the publisher.
